# Decadal variability in the occurrence of wintertime haze in central eastern China tied to the Pacific Decadal Oscillation

**DOI:** 10.1038/srep27424

**Published:** 2016-06-10

**Authors:** Sen Zhao, Jianping Li, Cheng Sun

**Affiliations:** 1 College of Global Change and Earth System Science (GCESS), Beijing Normal University, Beijing 100875, China; 2 Key Laboratory of Meteorological Disaster of Ministry of Education, Nanjing University of Information Science and Technology, Nanjing, China; 3 Joint Center for Global Change Studies, Beijing 100875, China

## Abstract

Haze is a serious issue in China with increasing concerns, and understanding the factors driving decadal-scale variations in haze occurrence is relevant for government policymaking. Using a comprehensive observational haze dataset, we demonstrate notable decadal fluctuations in the number of haze days (HD) during winter in central eastern China, showing a decline since the mid-1980s. The leading mode of the wintertime HD features an increasing trend for 1959–2012 in eastern China, highly correlated with China’s gross domestic product (GDP) that represents increasing trend of pollutant emissions, and to a lesser extent meteorological factors. The second mode shows decadal variations in central eastern China associated with Pacific Decadal Oscillation (PDO). Observations and numerical simulations suggest that Mongolia High and corresponding descending motion tend to be enhanced (weakened) in central eastern China during the positive (negative) phase of PDO. With PDO shifting towards a negative phase, the weakened Mongolia High and ascending anomalies make the air unstable and conduce to the spread of pollutants, leading to the decline in the wintertime HD over central eastern China since the mid-1980s. Based on above physical mechanisms, a linear model based on PDO and GDP metrics provided a good fit to the observed HD.

With the explosive economic development of China over the past few decades, haze pollution has become a serious environmental problem^1^,^2^,^3^, influencing human health^4^,^5^, visibility (potentially dangerous to air transportation and road traffic)^6^,^7^, and regional and global climate^8^,^9^. The number of haze days (HD) is an important indicator of the occurrence of haze pollution. In observation, a haze day is characterized by deteriorated atmospheric horizontal visibility of less than 10 km due to the fine particles at a relative humidity less than 80%^10^. The indicators of atmospheric aerosols (such as particle matter concentrations) are needed for further identification at a relative humidity between 80% and 95%^10^ (see Methods). The observed HD over eastern China has shown an increasing trend since the 1960s^11^,^12^,^13^,^14^. Previous studies^7^,^15^ indicated that this increasing trend of HD is closely related to increasing traffic and industrial emissions of pollutants associated with enhanced urbanization and industrialization. In addition to the air pollution due to the human activities, the meteorological conditions can also play an important role in the variability of haze^11^,^16^,^17^,^18^. Meteorological conditions as represented by a number of meteorological factors, such as winds, precipitation, atmospheric stability, and humidity can contribute to transportation, deposition, diffusion and transition of the haze particle, which leads to either maintenance or dispersion of haze^11^,^17^,^18^. The HD increasing trend over eastern China is highly correlated with the weakening of surface wind speed^14^ and decreasing trend of atmospheric relative humidity^11^. Several studies have suggested that the interannual variability of winter haze over China may be affected by the East Asian winter monsoon^19^ and the sea surface temperature (SST) in Atlantic Ocean^20^.

Previous studies^11^,^12^,^13^,^14^,^19^,^20^ about the HD variability in China mainly focus on the long-term trends and interannual variations, but the decadal-scale fluctuations in the HD have not been documented extensively. Figure 1a,b show observed linear trends in the wintertime (December to February, hereafter DJF) HD in China for both the earlier (1959/1960–1983/1984) and the recent (1984/1985–2012/2013) periods. The increasing trend in the HD over eastern China is evident in the earlier period (Fig. 1a). However, despite increasing traffic and industrial emissions, a significant decreasing trend is observed over central eastern China (26°N–38°N, 110°E–115°E) in the recent period (Fig. 1b). These changes to the HD are further illustrated by comparing linear trends for stations in central eastern China between the earlier and the recent periods (Fig. 1c). At most stations there is a slowing in the rise of the HD (36 stations; accounting for ~70% of total stations) or even a decline in the HD (22 stations; accounting for ~43% of total stations) since the mid-1980s (Fig. 1c). The averaged wintertime HD for those sites with slowing trend (36 stations) in central eastern China has shown a distinct downward trend since the mid-1980s and a decadal variation that closely follows the Pacific Decadal Oscillation (PDO) index (*r* = 0.66; *p* = 0.08; Fig. 1d), indicating that the wintertime HD in central eastern China is possibly related to the PDO.

The PDO^21^,^22^,^23^ is the dominant pattern of interdecadal variation in the Pacific SST. The PDO features an El Niño-like spatial pattern and corresponding time series exhibit considerable decadal variability^24^. PDO phase changes are accompanied by interdecadal variations in climatic conditions over many parts of the globe^25^. Previous studies^26^,^27^ have explicitly examined the relationships between the PDO and climate variations in China, such as winds, precipitation, and surface pressure. However, to our knowledge, there have been no studies that link decadal variations in haze weather to the PDO. An in-depth analysis of decadal variability of HD over eastern China and related meteorological factors are crucial to reveal the physical mechanisms between HD and the PDO and would have benefits for the HD prediction.

## Results

The dominant variability in the wintertime HD was extracted using an empirical orthogonal function (EOF) analysis over eastern China (south of 43°N and east of 105°E). The pattern of the first EOF (EOF1, Fig. 2a, referred to as the HD increasing mode), which accounts for 43.0% of the total variance, features an almost identical long-term trend in HD pattern over eastern China for the period 1959/1960–2012/2013. The EOF1 shows a strong spatial (pattern) correlation coefficient of 0.99 with the least-square linear trends of wintertime HD (Supplementary Fig. S1b). The trend pattern of wintertime HD is consistent with the observational results examined by previous studies^11^,^20^. The associated principal component (PC) (blue line in Fig. 2b) shows a marked increasing trend consistent with China’s GDP index (red line in Fig. 2b) with a high correlation coefficient of 0.96 (*p* = 0.05). GDP is a good proxy for industrialization and urbanization in China^28^,^29^. Along with the rapid industrialization and urbanization in China for 1960–2013, the emissions of carbon dioxide (CO_2_), nitrous oxide (N_2_O) (Supplementary Fig. S2a) and sulfur dioxide (SO_2_)^30^ increase with the GDP. This indicates that with rapid economic development, more pollutants are progressively released into the air, resulting in more HD. As expected, the most intensive increases in HD are found over the Yangtze River Delta, the Pearl River Delta, western Henan and Hubei provinces, and Beijing–Tianjin–Hebei (Supplementary Fig. S1), which have the fastest growing economies in China (Supplementary Fig. S2b, which also shows the locations of these regions).

**Figure 1:**
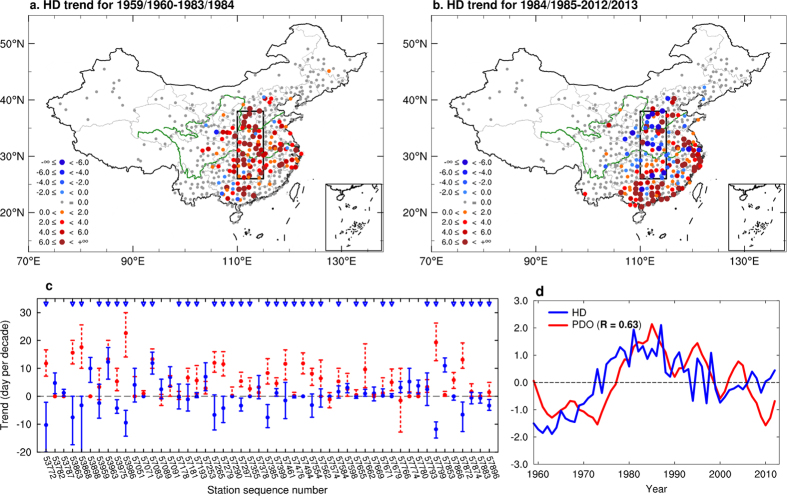
Observed regime change in the linear trend of the wintertime HD in eastern China for two periods: 1959/1960–1983/1984 (the earlier period) and 1984/1985–2012/2013 (the recent period). (**a,b**) Linear trend (days per decade) patterns of the wintertime HD in eastern China for the earlier period and the recent period, respectively. (**c**) Trends (circles) and 95% confidence limits (error bars) for the wintertime HD observed by the 51 stations (Supplementary Table S1) in central eastern China (26°N–38°N, 110°E–115°E, as indicated by the rectangle in (**a**,**b**)) for the earlier period (red) and the recent period (blue). Blue inverted triangles (36 stations, accounting for ~70% of the total number of stations) denote the stations for which the trends for the recent period are weaker than those for the earlier period. (**d**) Normalized central eastern China-averaged (36 stations averaged, see stations labeled with blue inverted triangles in (**c**)) wintertime HD time series (blue) and the PDO index (red) for 1959/1960–2012/2013. All plots were generated using NCAR Command Language (NCL). *Scientific Reports* remains neutral with regard to contested jurisdictional claims in published maps.

**Figure 2:**
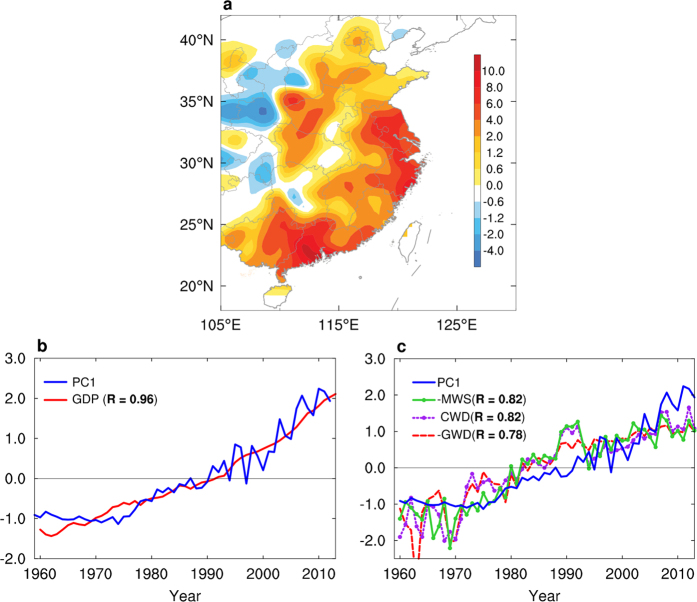
First principal mode of variability of the wintertime HD in eastern China, and relationships with GDP and surface wind. (**a**) Spatial pattern of the first EOF mode, which explains 43.0% of the variance. (**b**) First PC (PC1, blue) and the China GDP time series (red). (**c**) PC1 and time series of surface wind speed (MWS, green), calm wind days (CWD, purple), and gale wind days (GWD, red dotted line) averaged in central and eastern China. The MWS and GWD are multiplied by −1. Correlations coefficients between PC1 and GDP, MWS, CWD, and GWD are 0.96, 0.82, 0.82, and 0.78, respectively. All time series are normalized. All plots were generated using NCL. *Scientific Reports* remains neutral with regard to contested jurisdictional claims in published maps.

In addition to the substantial increase in pollutant emissions, contemporaneous changes are observed in meteorological conditions. Weakening surface winds over China have been reported previously^31^. Supplementary Fig. S3a–c show significant weakening trends in surface wind speed, significant increasing trends in the number of calm wind days, and slightly decreasing trends in the number of gale wind days over eastern China for the period 1959/1960–2012/2013. The areas with intensive increases in HD (east of 110°E) are well collocated with areas with strong wind decreases (Supplementary Fig. S3a). The PC1 time series has correlation coefficients of 0.82 (*p* = 0.05), 0.82 (*p* = 0.09), and 0.78 (*p* = 0.07) with China-averaged surface wind speed, number of calm wind days, and number of gale wind days, respectively (Fig. 2c). The weakening of wind speed is also evident in the lower troposphere (Supplementary Fig. S3d). The weaken advection effect of the winds is favourable to maintenance of haze over eastern China, thus contributes to the increasing of the wintertime HD. Significant enhanced trends in the atmospheric static stability (Supplementary Fig. S3g) and strengthened high pressure (Supplementary Fig. S3h) in the lower troposphere are evident over eastern China, which make the air more stable and hinder the spread of pollutants, and thus increase the HD. Consistent with result by Ding and Liu^11^, there is a significant reduced trend of relative humidity over eastern China (Supplementary Fig. S3i,j), indicating that reduced humidity may have inhibited the transition from haze to fog, resulting in an increase in the HD^11^. These changes in wind intensity and frequency, together with more stable stratification of the atmosphere and reduced atmospheric humidity, are conducive to the formation of haze weather and thus consistent with the increasing trend in HD over eastern China. However, neither the increasing pollutant burden nor the monotonic changes in climate conditions (Fig. 2c) can explain the decadal fluctuations in the HD in central eastern China, especially the decline in the HD since the mid-1980s (Fig. 1d).

**Figure 3:**
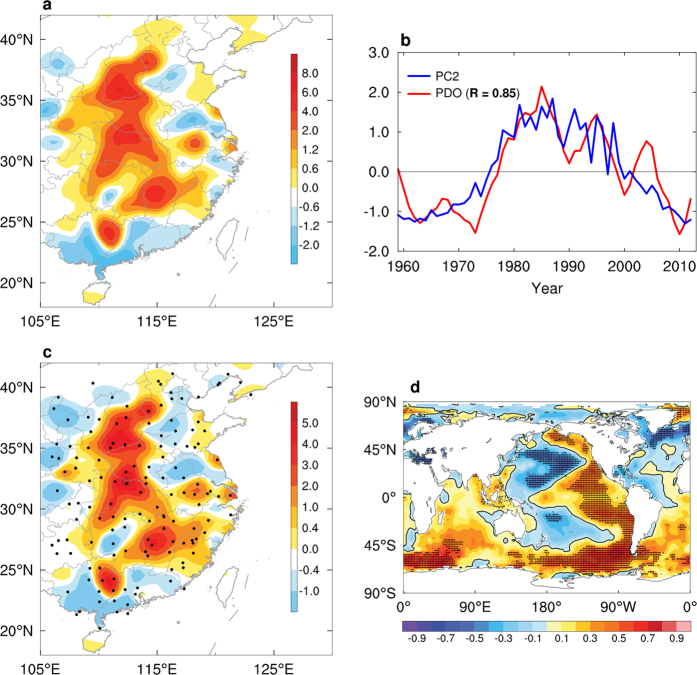
Second principal mode of variability of wintertime HD in eastern China, and relationships with the PDO and global SST. (**a**) Spatial pattern of the second EOF mode, which explains 14.1% of the variance. (**b**) The second PC (PC2, blue) and the PDO index (red) (*r* = 0.85). (**c**) Regression of the wintertime HD with the PDO index. (**d**) Correlation between annual mean global SST derived from ERSST and the PC2. Black dots indicate points for which the regression (in **c**) and correlation (in **d**) coefficients are significant at the 90% confidence level. All plots were generated using NCL. *Scientific Reports* remains neutral with regard to contested jurisdictional claims in published maps.

The second EOF (EOF2, Fig. 3a), which accounts for 14.1% of the total variance, features positive anomalies in central eastern China, flanked by weaker negative anomalies over the eastern coastal areas and western China (we refer to this as HD decadal mode). The PC2 time series (blue line in Fig. 3b) exhibits decadal variations with a switch from the most recent positive phase (1977–1998) to the most recent negative phase (1999–2012), strongly in phase with the PDO index (red line in Fig. 3b) with a correlation coefficient of 0.85 (*p* = 0.07). Regression patterns of wintertime HD anomalies with respect to the PDO show significant positive anomalies in central eastern China (Fig. 3c) and largely resemble the EOF2 pattern. These results are also supported by using linearly detrended wintertime HD anomalies (with linear trends subtracted) during the same period (Supplementary Fig. S4). Furthermore, the regression pattern of annual-mean global SST anomalies with respect to the PC2 time series features a PDO-like SST pattern (Fig. 3d). This relationship is not sensitive to the choice of SST data set (Supplementary Fig. S5), indicating that decadal variations in wintertime HD in central eastern China are significantly related to the PDO.

To understand the underlying mechanism of the relationship between the PDO and the HD decadal mode, we first examine the major meteorological factors that are linked to HD on decadal timescales. Supplementary Fig. S6 presents the correlations between the PC2 and winds, precipitation, atmospheric stability, and relative humidity. The correlations of the winds (Supplementary Fig. S6a–d), precipitation (Supplementary Fig. S6e,f), atmospheric static stability (Supplementary Fig. S6g), and relative humidity (Supplementary Fig. S6i,j) with the HD decadal mode are relatively low, indicating that the advection effect of winds, the washout effect of precipitation, and the transition between fog and haze might not be the important processes responsible for the HD over central eastern China at the decadal timescale. Saha^32^ and Zheng *et al*.^18^ reported that geopotential height and vertical velocity fields are the important proxies for the dynamical stability, which are effective indicators to identify the diffusion of air pollutants. As shown in Supplementary Fig. S6h, it is found that the HD decadal mode is significantly correlated with 850 hPa geopotential height over eastern China, indicating that the dynamical stability may play an important role in determining the HD decadal variations.

**Figure 4:**
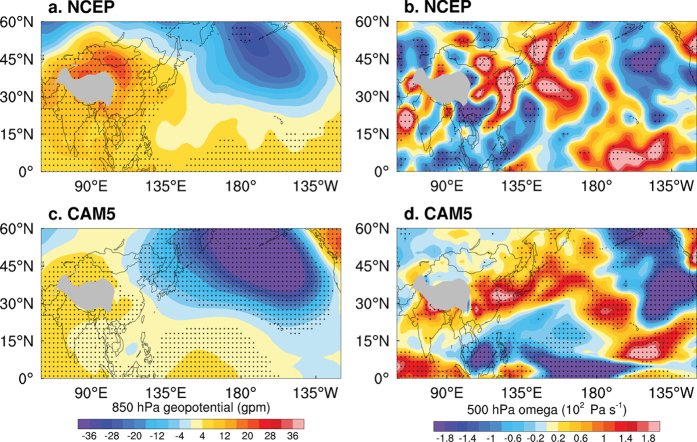
Observed and simulated atmospheric circulation patterns associated with PDO decadal fluctuations. Composite differences of 850 hPa geopotential height (**a**) and 500 hPa vertical velocity (**b**) for high and low values of the PDO index from the NCEP/NCAR Reanalysis (dots indicate differences that are significant at the 90% confidence level). (**c**) and (**d**) as for (**a**) and (**b**), respectively, but for the CAM5 simulations. Grey areas indicate the Tibetan Plateau. All plots were generated using NCL.

To gain better insight into the potential influence of the PDO on decadal changes in the HD, Fig. 4a,b show the differences between 850 hPa geopotential height and 500 hPa vertical velocity for composite periods of high and low PDO based on the NCEP/NCAR reanalysis. The wintertime climatological circulation over East Asia in the lower troposphere is dominated by the cold high, with its center around Mongolia (Mongolia High)^33^. It is suggested that the simultaneous variation between the Aleutian Low and the Mongolian High could be an important way by which the PDO exerts its impact on the atmospheric circulation over East Asia^26^. During the positive phase of the PDO, the Mongolia High significantly strengthens and moves southward, resulting in a high pressure across the eastern China (Fig. 4a). The high pressure is accompanied by strongly sinking motion in central eastern China (Fig. 4b). The wintertime mean planetary boundary layer (PBL) height is about 1–1.5 km over eastern China^34^. The large-scale sinking motion occurs mostly above 800 hPa (about 2 km) (Supplementary Fig. S7a), it prevents pollutants in the PBL to be mixed upward into the free troposphere, and it also does not help lateral dispersion because the divergence does not reach the ground (large divergence anomaly occurs above 850 hPa) in central eastern China (Supplementary Fig. S7b). These results suggest that the high pressure and sinking motion above the PBL in central eastern China makes the surface layer more stable and hampers vertical mixing of pollutants, consistently resulting in more HD over central eastern China. Supplementary Fig. S8a,b show the divergence anomalies in the lower and convergence anomalies in the upper troposphere in central eastern China, which are favourable to the maintenance of sinking motion. The composite difference patterns (Fig. 4a) are similar to the 500 hPa height patterns shown by Zhang *et al*.^22^ and Dong *et al*.^35^, although the time periods of the chosen positive and negative PDO phases are slightly different. Consistent results can be obtained by removing the linear trend in atmospheric circulation (Supplementary Fig. S9). The strengthened Mongolia High during the positive phase of the PDO is also evident in the sea-level pressure derived from NCEP/NCAR reanalysis and in observations of station surface pressure (Supplementary Fig. S10). These results indicate a clear tendency towards increased (decreased) occurrences of hazy weather in central eastern China during the positive (negative) phase of the PDO.

To assess the extent to which the PDO influences atmospheric circulation in China, sensitivity experiments were conducted using the Community Atmosphere Model version 5 (CAM5) atmospheric general circulation model (AGCM). Composite SST anomalies for high- and low-PDO periods in the Pacific Ocean basin were prescribed in the model (Supplementary Fig. S11), characterized by a horseshoe pattern in the Pacific, meridionally broader than the ENSO. The AGCM experiment performed well in qualitatively reproducing the observed intensification and southward moving of the Mongolia High, corresponding descending motion in response to PDO forcing (Fig. 4c,d) and the divergence anomalies in the lower and convergence anomalies in the upper troposphere in central eastern China (Supplementary Fig. S8c,d).

**Figure 5:**
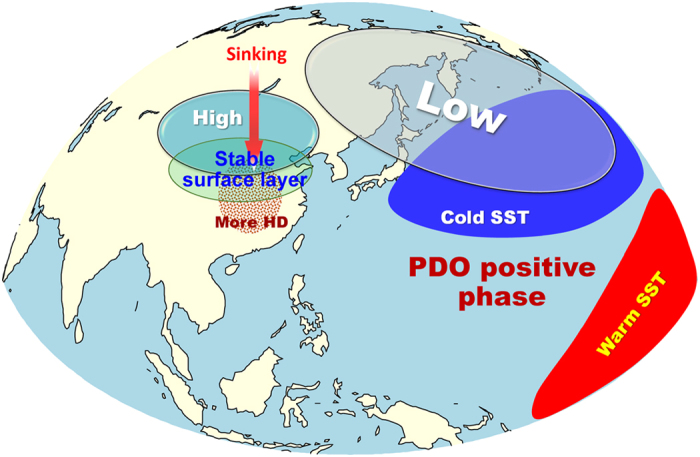
A schematic diagram of the influence of the PDO on the wintertime HD in central eastern China. Low, Aleutian Low anomalies; High, Mongolian High anomalies. This plot was generated using NCL and licensed Microsoft PowerPoint.

Overall, our results provide strong evidence that in recent decades the PDO has played an important role in modulating the wintertime HD in central eastern China. Figure 5 shows a schematic diagram of the processes that summarizes the influence of the PDO on the wintertime HD in central eastern China. During the positive phase of the PDO, the Aleutian Low strengthens and extends westward, and the Mongolian High strengthens and moves southward, resulting high pressure and sinking anomalies in central eastern China. The high pressure and sinking motion above the PBL makes the surface layer more stable and hampers vertical mixing of pollutants, leading to more HD in central eastern China. With the PDO shifting towards the most recent negative phase (1999–2012), our results suggest that the Mongolia High and corresponding descending motion tend to be weakened, which make the air unstable and conduce to the spread of pollutants, thus leading to the decline in the wintertime HD over central eastern China since the mid-1980s (Fig. 1d).

## Discussion

**Figure 6:**
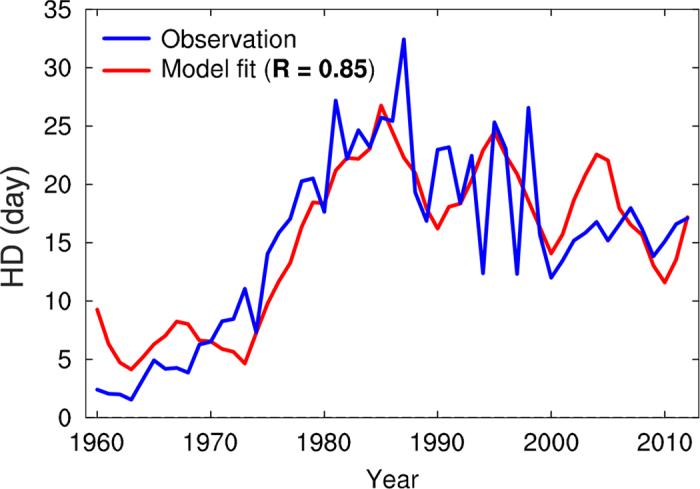
The observed and modeled wintertime HD in central eastern China. Observed average (22 stations; see Supplementary Fig. S12) wintertime HD time series in central eastern China from 1960/1961 to 2012/2013 (blue), and the linear model (Equation (1)) fitted to the data (red). This plot was generated using NCL.

Our findings suggest that decadal variability in the wintertime HD is closely tied to the PDO, and this has implications for decadal predictions of the HD in central eastern China. In order to focus on the role of the PDO, and considering the complexity of its impacts, only stations that were found to have a significant positive correlation with the PDO were selected for further modeling and analysis (Supplementary Fig. S12a). An empirical linear model for central eastern China was developed to predict the average HD in winter based on China’s GDP and the PDO, as follows:


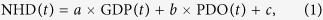


where *t* is time in years and the parameters *a* = 9.8, *b* = 2.8, and *c* = 15.4 were determined empirically by linear regression, so that the regression error of Eq. (1) was minimized. As shown in Fig. 6, the linear model fitted to the HD data closely follows the observed HD (*r* = 0.85; *p* = 0.01). The performance of the empirical linear model is largely insensitive to the selection of stations in central eastern China, the averaged result obtained from 36 stations is provided in Supplementary Fig. S12b. This result indicates that rigorously determined decadal predictions of Pacific SSTs associated with the PDO, for example from initialized decadal forecasts using coupled GCMs, could produce improved decadal predictions of the HD in central eastern China.

It should be noted that the HD exhibits considerable variability at the interannual timescale, and the empirical linear model show biases in capturing the HD variations in the mid-2000s (Fig. 6), suggesting that the PDO is not the only unique driver for the HD variability and other processes may also play a role in modulating it. Despite the relatively low predictive skill of the PDO index in CMIP5 coupled models, recent studies^36^,^37^ have shown that an initialization of the upper-ocean state using historical observations is effective for successful hindcasts of the PDO and may have a large impact on future predictions. Over the coming decades, a predicted tendency towards a negative PDO phase^36^,^38^ may suppress the rising trend in the wintertime HD as predicted from the increased pollutant burden in central eastern China.

## Methods

### Haze observations

The haze dataset was derived from data provided by the National Meteorological Information Center of the China Meteorological Administration (CMA) based on observations taken across a network of 2474 meteorological stations between 1954 and 2013, in accordance with their strict quality control procedures and protocols. Haze days, as defined by the CMA^10^, occur when visibility is less than 10 km (after excluding rain, dust storms, and other weather phenomena that affect visibility) and when the daily mean relative humidity is less than 80%. When the daily mean relative humidity is between 80% and 95%, further identification^26^ is needed by considering the specifications of surface meteorological observations or indicators of atmospheric aerosols. The monthly HD can be calculated provided no missing records occur for an entire month at any given station (as was the case for the 756 stations displayed in Supplementary Fig. S13a). The period 1960–2013 and the winter season (DJF) were chosen since during this period and season, contiguous HD records exist for many stations (Supplementary Fig. S13b) and the occurrence of haze days reaches a peak during the boreal winter in China (Supplementary Fig. S14b). Stations featuring record gaps amounting to more than 0.01% of the total record for 1960–2013 (i.e., more than 6 monthly records over 648 months) were excluded from the analysis, as were stations with all zero values (Supplementary Fig. S15). As a result, 480 weather stations with wintertime HD records for 54 years (1959/1960–2012/2013) were selected (Supplementary Fig. S15).

### Meteorological conditions observations

Daily observations of 10 m wind speed, surface pressure, and relative humidity for the 480 stations (Supplementary Fig. S14) were also obtained. **Calm wind days** are defined as days where the maximum wind speed at 10 m is less than 3 m s^−1^. **Gale wind days** are defined as days where the maximum wind speed at 10 m is greater than 17 m s^−1^.

### Reanalysis and gridded observation data set

The National Centers for Environmental Prediction–National Center for Atmospheric Research (NCEP/NCAR) dataset was used for reanalysis^39^. The gridded global SST was obtained from the NOAA Extended Reconstructed Sea Surface Temperature (ERSST) V3b^40^, the Met Office Hadley Centre’s sea surface temperature (HadISST)^41^, and HadSST3^42^,^43^.

### GDP

The GDP in China during 1960–2013 was obtained from the World Bank (from http://databank.worldbank.org/data/home.aspx, accessed July 2015) to represent the economic growth and accelerating urbanization in China based on their bidirectional causality^29^. The logarithm of GDP from the original time series was used to eliminate the influence of heteroscedasticity and drastic fluctuations in the economic data. We also used the gross regional product for 1993–2013 obtained from the National Bureau of Statistics of China (from http://data.stats.gov.cn/, accessed July 2015), as presented in Supplementary Fig. S2b.

### PDO index

The monthly PDO index for 1957 to 2014 was obtained from the University of Washington (from http://jisao.washington.edu/pdo/PDO.latest, accessed July 2015). Details of this index are provided by Mantua *et al*.^21^. To focus on the decadal variations, the raw index is first annually averaged and then the time series is smoothed by a 5-year running-mean.

### Statistical methods

EOF analysis^44^ was performed to identify the wintertime HD for the 355 stations over eastern China (south of 43°N and east of 105°E) that that haze weather mainly occurred (Supplementary Fig. S14a). EOF1 and EOF2 are well separated each other and from other higher modes based on the criteria suggested by North *et al*.^45^. Trends for HD were calculated as the Sen median slope^46^ and tested for statistical significance using the Mann–Kendall test. The significance of regression and correlation coefficients were tested using a two-tailed Student’s *t*-test, accounting for temporal autocorrelation using the effective sample size^47^,^48^,^49^. The effective sample size N^*^ can be estimated by the following approximation:


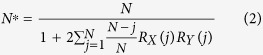


where *N* is the number of available time steps, and *R*_*X*_(*j*) and *R*_*Y*_(*j*) are the autocorrelations of two sampled time series *X* and *Y* at time lag *j*. Student’s *t*-test was used to test the significance of composited differences.

### CAM5 model simulations

The Community Atmosphere Model version 5 (CAM5), developed at NCAR, was used to verify the teleconnection link and to establish the direction of the causal relationship between PDO and the East China regional climate. CAM5 is the atmospheric component of the Community Earth System Model, CESM 1.0.4. The finite-volume dynamical core of CAM5 was used with a global horizontal resolution of ~2°. The Community Land Model and the thermodynamic module of the Community Sea-Ice Model were used to estimate the heat flux at the surface, to ensure that the SST was the only external lower-boundary forcing on the atmospheric model.

A control run was forced with monthly varying, annually repeating SST and sea-ice concentration, and carbon dioxide and ozone concentrations, all fixed at 1980s levels. Two forced runs were established by changing only the SSTs in the Pacific basin. The SST anomalies were imposed following the PDO composite SST pattern for the periods of high and low PDO index. The difference between two forced runs defined the response of the atmospheric model to the PDO SST forcing. All integrations were run for 35 years starting from the same initial conditions, and the results from years 5 to 35 were used to investigate the model response to the specified SST forcing.

### Graphic software

All maps and plots were produced using NCAR Command Language (NCL, ref. 50), while Fig. 5 was additionally modified by licensed Microsoft PowerPoint.

## Additional Information

**How to cite this article**: Zhao, S. *et al*. Decadal variability in the occurrence of wintertime haze in central eastern China tied to the Pacific Decadal Oscillation. *Sci. Rep.* **6**, 27424; doi: 10.1038/srep27424 (2016).

## Supplementary Material

Supplementary Information
