# Supplementary material for: Decadal variability in the occurrence of wintertime haze in central eastern China tied to the Pacific Decadal Oscillation
Source: Sci Rep. 2016 Jun 10;6:27424. doi: 10.1038/srep27424 (PMC4901280; doi:10.1038/srep27424)
Supplement: Supplementary Information [file srep27424-s1.pdf]

Supplementary Information for

Decadal variability in the occurrence of wintertime haze in central eastern China tied to the Pacific Decadal Oscillation

Sen Zhao,[1,4] Jianping Li,[2,3,*] and Cheng Sun[2,3,*]

1. State Key Laboratory of Numerical Modeling for Atmospheric Sciences and Geophysical Fluid Dynamics, Institute of Atmospheric Physics, Chinese Academy of Sciences, Beijing 100029, China
2. College of Global Change and Earth System Science (GCESS), Beijing Normal University, Beijing 100875, China
3. Joint Center for Global Change Studies, Beijing 100875, China
4. University of Chinese Academy of Sciences, Beijing 100049, China

*Correspondence and requests for materials should be addressed to J.L. (email: ljp@bnu.edu.cn) or C.S. (email: scheng@bnu.edu.cn)

This file includes Supplementary Table S1 and Supplementary Figs. S1–S15, which can be classified as follows:

(1) Meteorological stations information

Supplementary Table S1 shows the detailed information of the 51 stations in central eastern China. Supplementary Figs. S13-S15 show the information and selection of meteorological stations for wintertime number of haze days (HD) records.

(2) The HD increasing mode and its physical mechanism

The HD increasing mode features almost identical spatial pattern with the long-term linear trends of wintertime HD as shown in Supplementary Fig. S1. Supplementary Figs. S2 and S3 investigate the relationships of HD increasing mode with human activities and meteorological factors, respectively.

(3) The HD decadal mode and its physical mechanism

Supplementary Fig. S4 shows the robustness of the HD decadal mode by removal of the linear trends. Supplementary Fig. S5 show the relationship between the HD decadal mode and annual mean global SST using HadISST and Kaplan datasets. Supplementary Fig. S6 investigates the relationships between HD decadal mode and meteorological factors. Supplementary Fig. S7 investigates the vertical profile of vertical motion and divergence in

central eastern China associated with the PDO. Supplementary Fig. S8 investigates the relationships between the PDO and convergence and divergence. Supplementary Figs. S9 and S10 conform the relationship between the HD decadal mode and atmospheric dynamical stability by removal of linear trends, or by using sea level pressure and station pressure. Supplementary Fig. S11 shows the prescribed SST anomalies of the experiments in CAM5 simulations.

(4) Empirical linear model

Supplementary Fig. S12 suggests the empirical linear model is largely insensitive to the selection of stations in central eastern China.

Supplementary Table S1

Supplementary Table S1. List of the 51 stations in central eastern China for which data are presented in Figure 1**c**.

Station sequence number	Province	Station name	Latitude	Longitude	Elevation
53772	Shanxi	Taiyuan	37.52	112.33	776.3
53782	Shanxi	Yangquan	37.55	113.30	767.2
53787	Shanxi	Yushe	37.04	112.59	1041.4
53863	Shanxi	Jiexiu	37.02	111.54	743.9
53868	Shanxi	Linfen	36.05	111.32	449.5
53898	Henan	Anyang	36.08	114.21	62.9
53959	Shanxi	Yuncheng	35.02	111.00	365.0
53963	Shanxi	Houma	35.39	111.22	433.8
53975	Shanxi	Yangcheng	35.29	112.24	659.5
53986	Henan	Xinxiang	35.21	113.50	73.2
57051	Henan	Sanmenxia	34.48	111.11	409.9
57071	Henan	Mengjin	34.50	112.26	333.3
57083	Henan	Zhengzhou	34.46	113.39	110.4
57089	Henan	Xuchang	34.01	113.49	67.2
57091	Henan	Kaifeng	34.50	114.20	73.7
57178	Henan	Nanyang	33.04	112.32	129.2
57181	Henan	Baofeng	33.53	113.03	136.4
57193	Henan	Xihua	33.45	114.30	52.6
57253	Hubei	Yunxian	32.48	110.45	201.9
57265	Hubei	Laohekou	32.25	111.40	132.1
57279	Hubei	Zaoyang	32.09	112.40	125.5
57290	Henan	Zhumadian	32.58	114.03	82.7
57297	Henan	Xinyang	32.10	114.05	114.5
57355	Hubei	Badong	31.04	110.24	334.0
57378	Hubei	Zhongxiang	31.10	112.34	65.8
57385	Hubei	Guangshui	31.37	113.59	93.3
57399	Hubei	Macheng	31.11	114.58	74.3
57461	Hubei	Yichang	30.42	111.05	133.1
57476	Hubei	Jingmen	30.24	112.04	31.8
57494	Hubei	Wuhan	30.38	114.17	23.6
57554	Hunan	Sangzhi	29.24	110.08	322.2
57562	Hunan	Shimen	29.36	111.23	116.9
57574	Hunan	Nanxian	29.20	112.25	36.0
57584	Hunan	Yueyang	29.23	113.05	53.0
57598	Jiangxi	Xiushui	29.02	114.34	146.8
57655	Hunan	Yuanling	28.27	110.23	151.6

Station sequence number	Province	Station name	Latitude	Longitude	Elevation
57662	Hunan	Changde	28.55	111.33	150.6
57669	Hunan	Anhua	28.28	111.18	128.3
57671	Hunan	Yuanjiang	28.51	112.22	37.0
57679	Hunan	Mapoling	28.15	112.50	115.0
57766	Hunan	Shaoyang	27.15	111.23	311.0
57774	Hunan	Shuangfeng	27.28	112.11	100.0
57780	Hunan	Zhuzhou	27.50	113.09	74.6
57793	Jiangxi	Yichun	27.48	114.23	131.3
57799	Jiangxi	Ji'anxian	27.05	114.55	71.2
57853	Hunan	Wugang	26.44	110.37	341.0
57866	Hunan	Yongzhou	26.22	111.31	172.6
57872	Hunan	Hengyang	26.56	112.30	104.9
57874	Hunan	Changning	26.26	112.17	116.6
57883	Jiangxi	Xiaping	26.50	114.00	253.0
57896	Jiangxi	Suichuan	26.15	114.20	126.1

Supplementary Fig. S1

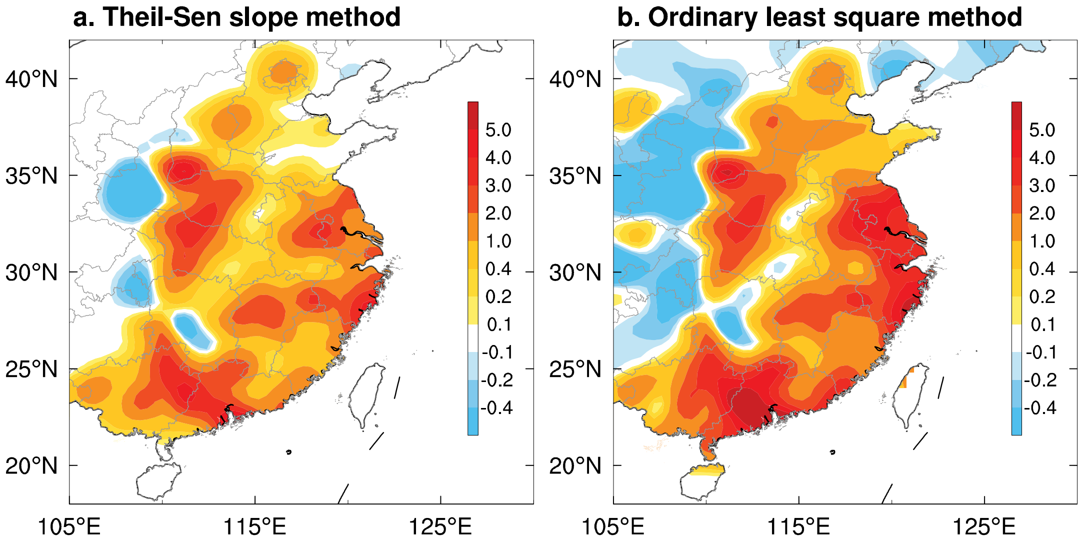

Supplementary Fig. S1. Long-term linear trend (days per decade) of the wintertime HD in eastern China for 1959/1960 to 2012/2013, as calculated using the Theil-Sen median slope method (**a**) and the ordinary least squares method (**b**) (see Methods). The spatial correlation coefficients (EOF1) for 355 stations are 0.88 and 0.99, respectively. All plots were generated using NCAR Command Language (NCL). *Scientific Reports* remains neutral with regard to contested jurisdictional claims in published maps.

Supplementary Fig. S2

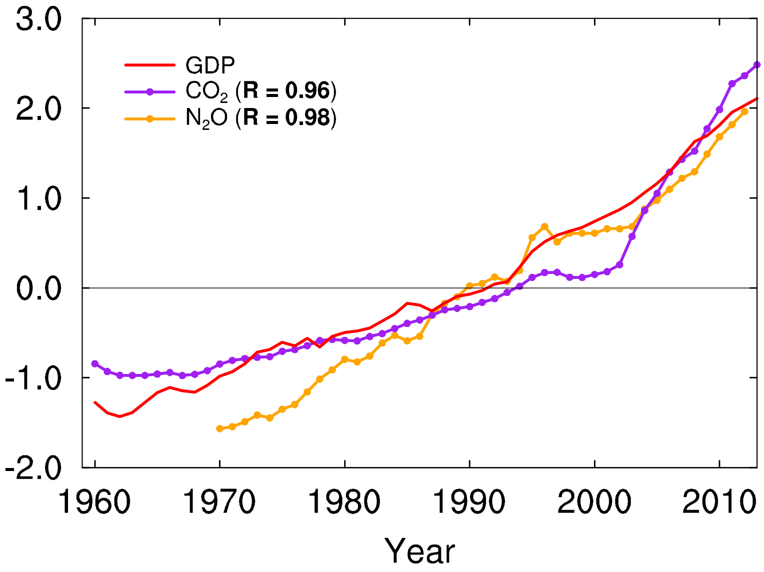

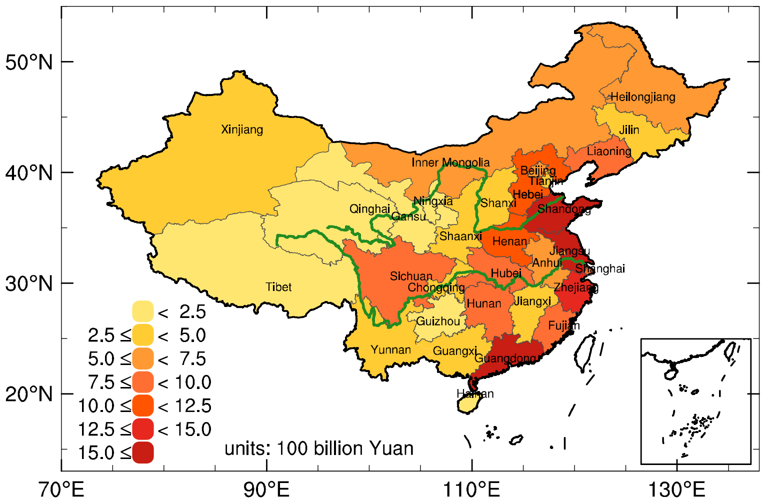

Supplementary Fig. S2. **a,** Normalized China GDP time series (*red*), CO_2 emissions in China derived from Global Carbon ATLAS (*purple*), and N_2O emissions derived from the Emissions Database for Global Atmospheric Research (*orange*). **b**, Long-term mean of the gross regional product (GRP) in the Chinese mainland for 1993–2013. All plots were generated using NCL. *Scientific Reports* remains neutral with regard to contested jurisdictional claims in published maps.

Supplementary Fig. S3

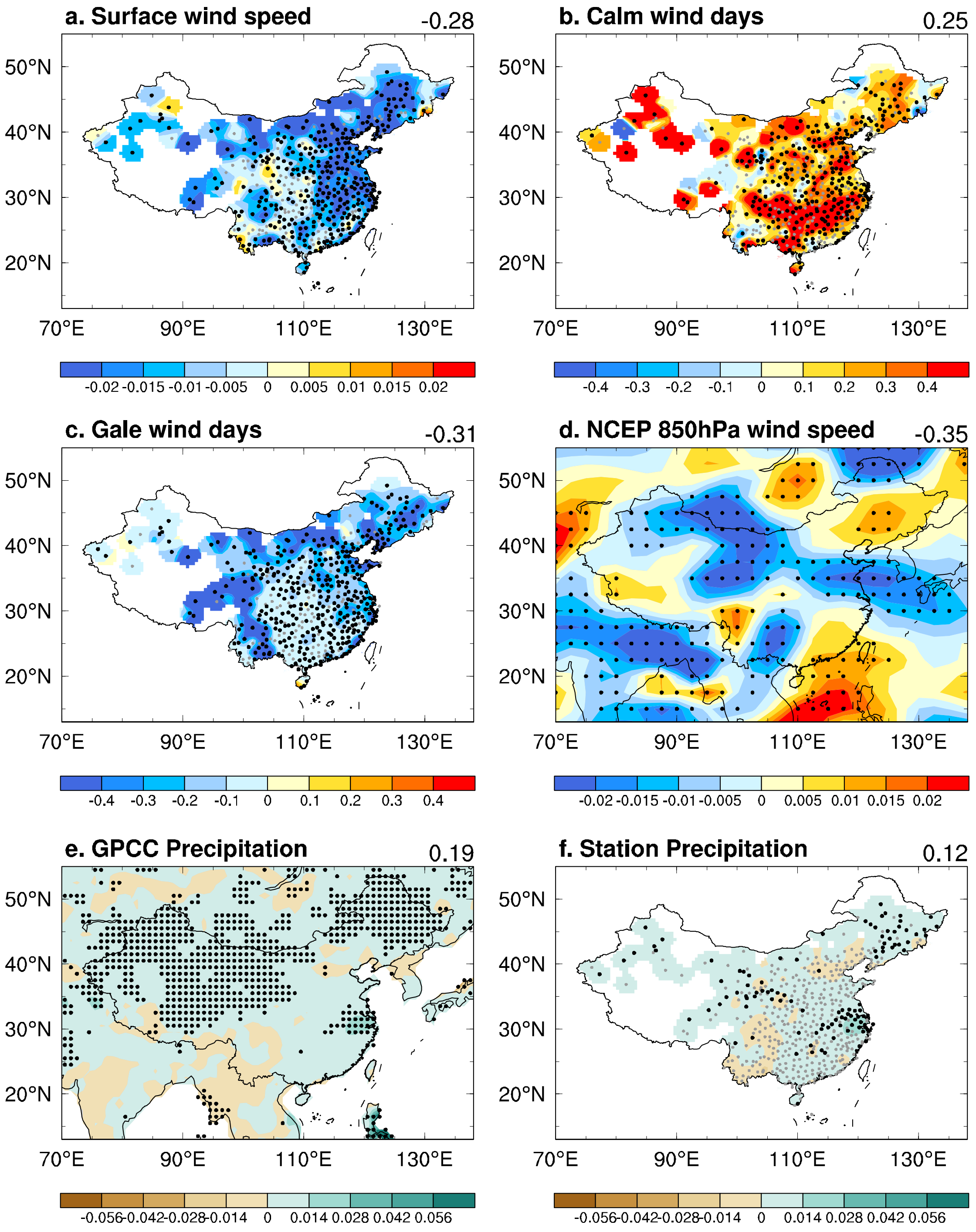

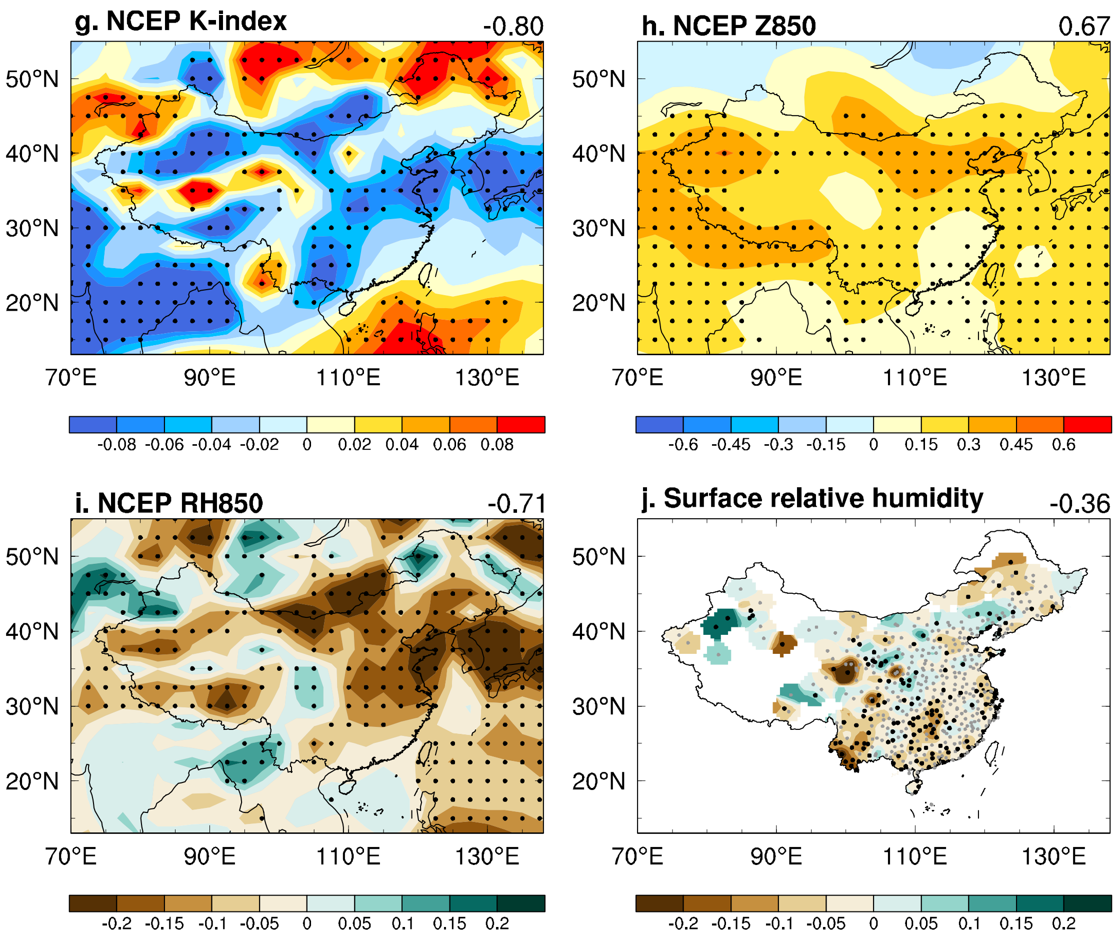

Supplementary Fig. S3. Long-term linear trend of the wintertime meteorological factors for 1959/1960 to 2012/2013. **a**, mean surface wind speed (m s^{-1} a^{-1}); **b**, number of calm wind days (day a^{-1}); **c**, number of gale wind days (day a^{-1}); **d**, 850 hPa wind speed (m s^{-1} a^{-1}); **e**, GPCC precipitation (mm a^{-1}); **f**, station precipitation (mm a^{-1}); **g**, K-index (K a^{-1}); **h**, 850 hPa geopotential height (m a^{-1}); **i**, 850 hPa relative humidity (a^{-1}); **j**, surface relative humidity (a^{-1}). (**a**, **b**, **c**, **f**, **j**) are derived from the station datasets, (**d**, **g**, **h**, **i**) are derived from NCEP reanalysis. Numbers in top right of panels separately denote the spatial pattern correlation coefficients with the HD trend. Calm wind days are defined as days where the maximum wind speed at 10 m is less than 3 m s^{-1}. Gale wind days are defined as days where the maximum wind speed at 10 m is greater than 17 m s^{-1}. The K-index is used to represent the stability of atmospheric stratification and is defined as $(T_{850} - T_{500}) + Td_{850} + (T - Td)_{700}$, where Ti is the air temperature at the ith level and $(Td)j$ is the dew point temperature at the jth level, which is estimated from the relative humidity and the actual air temperature based on the Magnus–Tetens approximation. The negative K-index anomalies mean more stable of the atmospheric stratification. Trends were calculated using the the ordinary least squares method. The black (gray) dots denote the locations of stations or grids with significant (insignificant) correlations at the 90% confidence level. All plots were generated using NCL. *Scientific Reports* remains neutral with regard to contested jurisdictional claims in published maps.

Supplementary Fig. S4

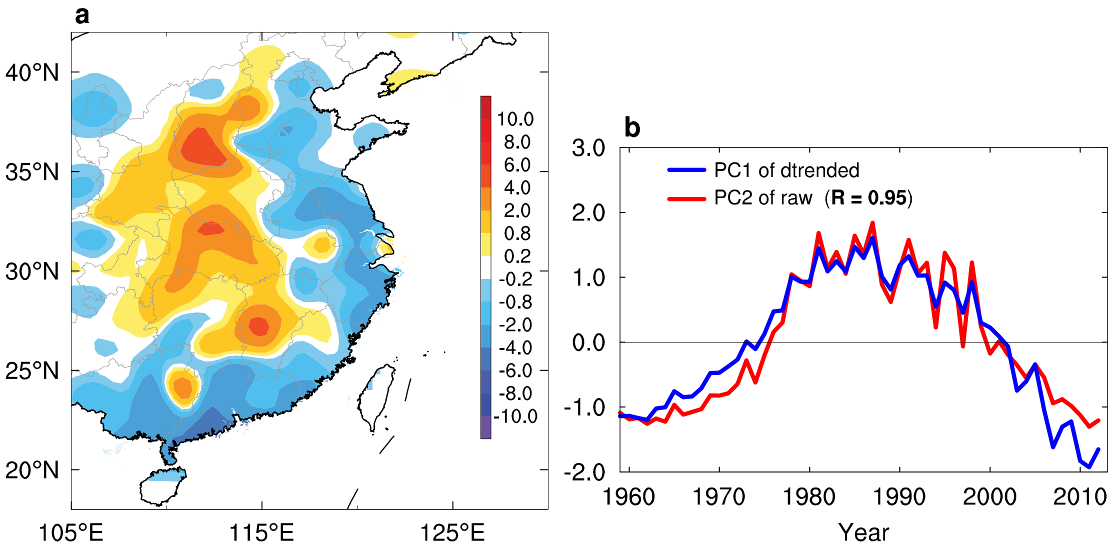

Supplementary Fig. S4. a, The leading mode of variability of the detrended wintertime HD in eastern China, and **b**, relationships with the PC2 in Figure 3**b**. All plots were generated using NCL. *Scientific Reports* remains neutral with regard to contested jurisdictional claims in published maps.

Supplementary Fig. S5

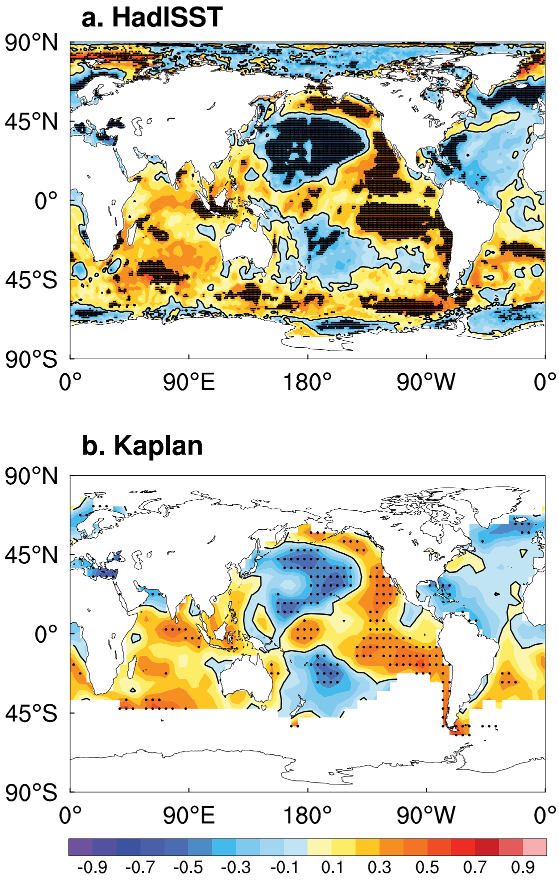

Supplementary Fig. S5. Correlations of annual mean global SST derived from HadISST (a) and Kaplan (b) with the PC2 of HD during 1959–2012. Dots indicate that correlations are significant at the 90% confidence level. All plots were generated using NCL.

Supplementary Fig. S6

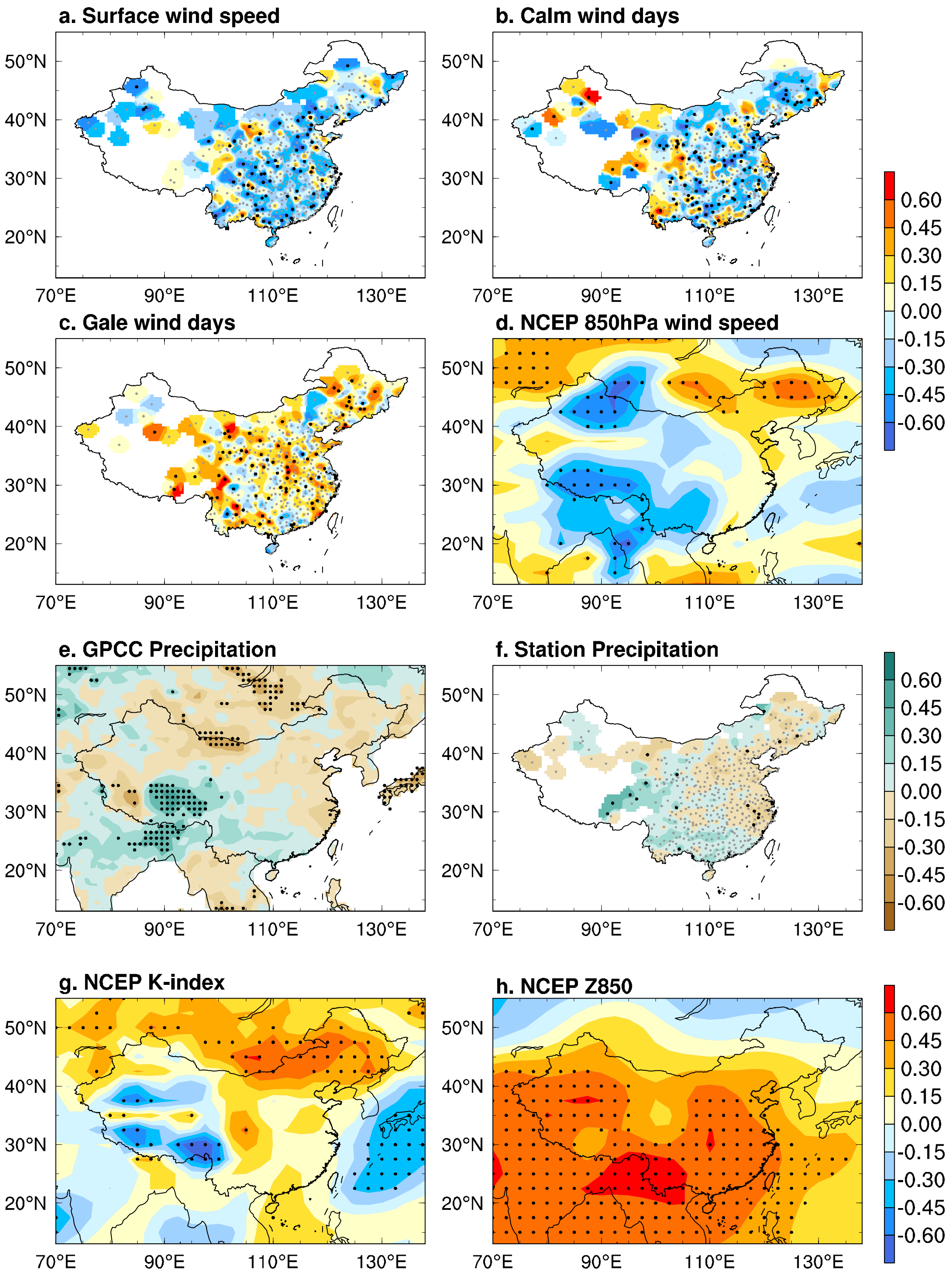

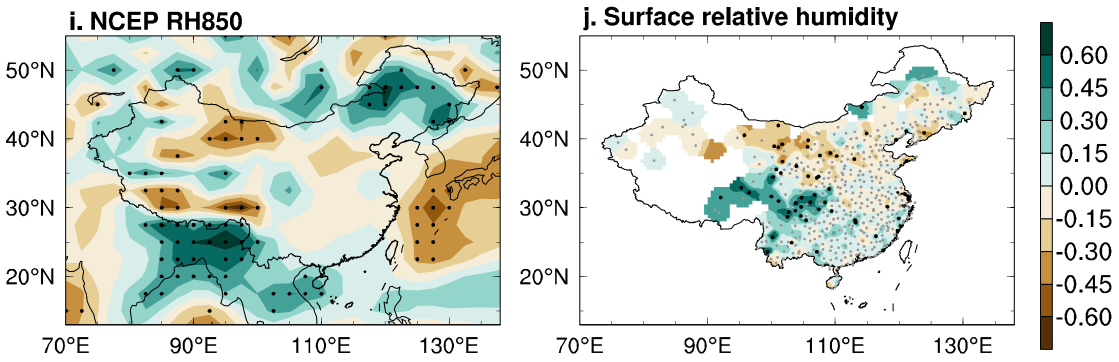

Supplementary Fig. S6. Spatial distributions of the correlation coefficients between the PC2 of HD and wintertime mean surface wind speed (**a**), number of calm wind days (**b**), number of gale wind days (**c**), 850 hPa wind speed (**d**), GPCC precipitation (**e**), station precipitation (**f**), K-index (**g**), 850 hPa geopotential height (**h**), 850 hPa relative humidity (**i**) and surface relative humidity (**j**) for 1959/1960–2012/2013. The black (gray) dots denote the locations of stations or grids with significant (insignificant) correlations at the 90% confidence level. All plots were generated using NCL. *Scientific Reports* remains neutral with regard to contested jurisdictional claims in published maps.

Supplementary Fig. S7

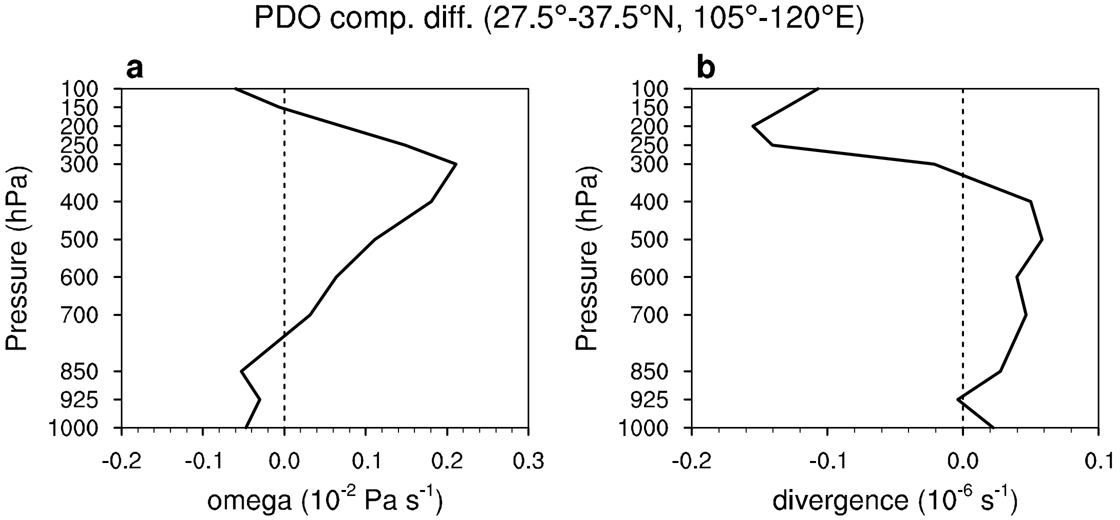

Supplementary Fig. S7. Composite differences of regional-averaged vertical velocity (**a**) and divergence (**b**) profiles over central eastern China (27.5°-37.5°N, 105-120°E) between positive and negative PDO phases. All plots were generated using NCL.

Supplementary Fig. S8

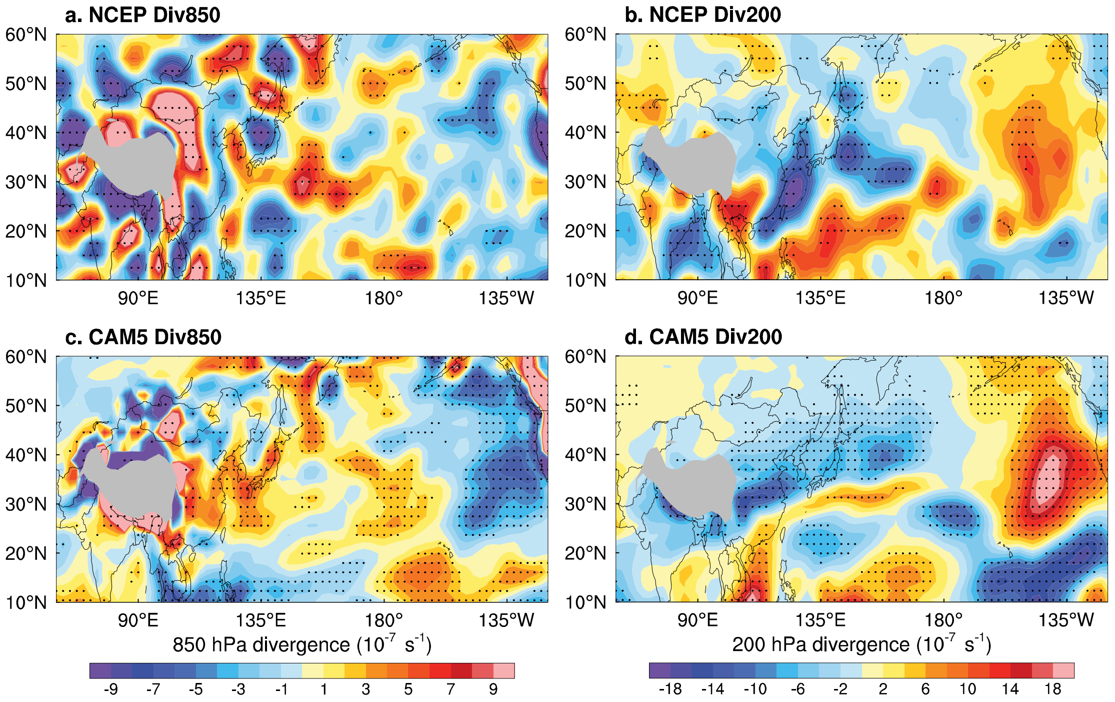

Supplementary Fig. S8. Composite differences of divergence at 850 hPa (**a**) and 200 hPa (**b**) for high and low values of the PDO index from the NCEP/NCAR Reanalysis. **c** and **d** as for **a** and **b**, respectively, but for the CAM5 simulations. Grey areas indicate the Tibetan Plateau. All plots were generated using NCL.

Supplementary Fig. S9

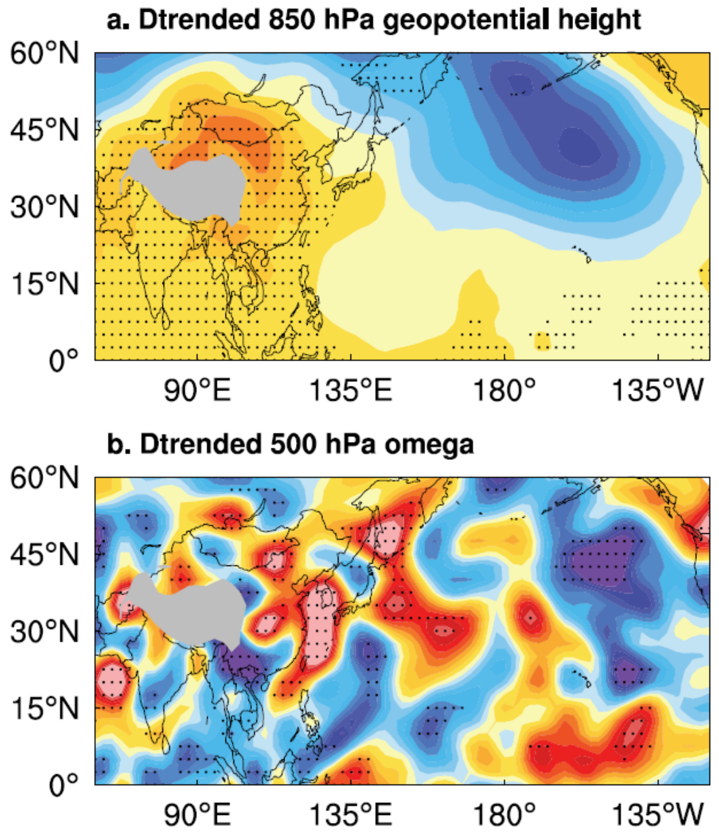

Supplementary Fig. S9. a and **b** same as Figure 4**a** and 4**b**, respectively, but with linear trends removed. All plots were generated using NCL.

Supplementary Fig. S10

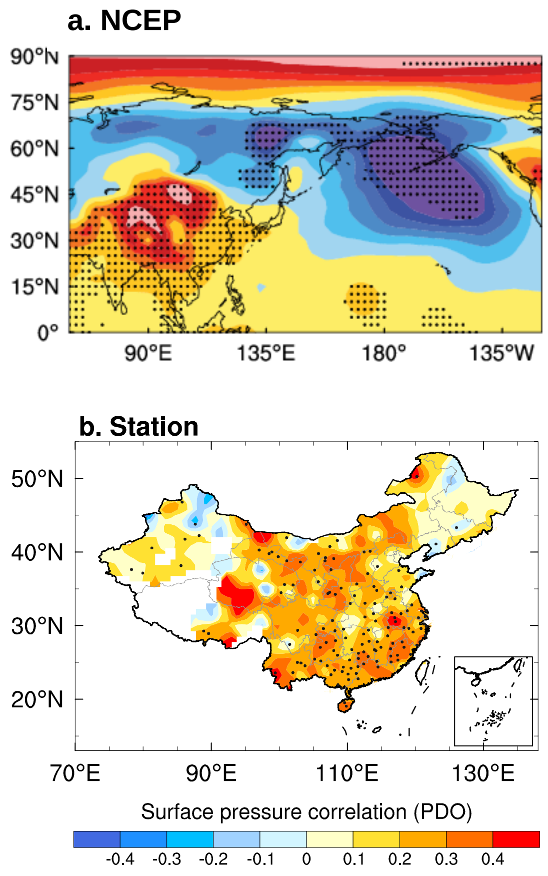

Supplementary Fig. S10. a, Regressions of wintertime sea level pressure derived from NCEP reanalysis with the PDO for 1959/1960–2011/2012. **b**, Correlations of wintertime surface pressure from observation with the PDO for 1959/1960–2011/2012. Dots indicate that correlations are significant at the 90% confidence level. All plots were generated using NCL. *Scientific Reports* remains neutral with regard to contested jurisdictional claims in published maps.

Supplementary Fig. S11

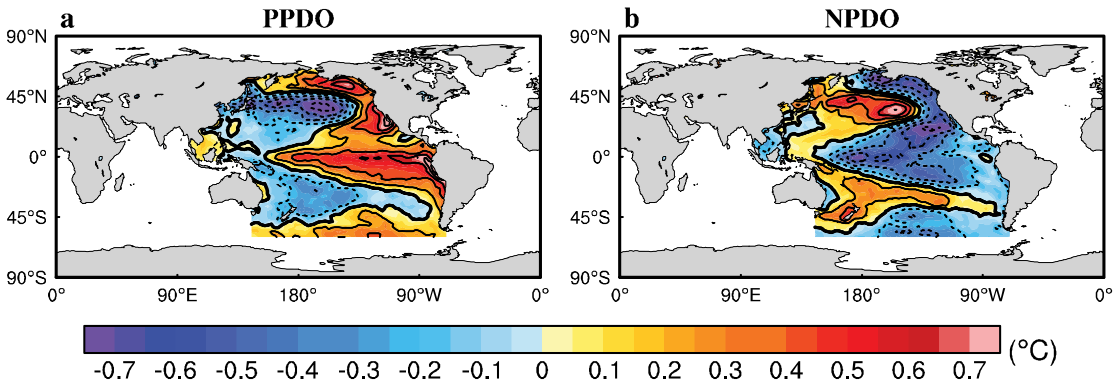

Supplementary Fig. S11. Prescribed SST anomalies of the experiments in CAM5 simulations. The SST anomalies are based on the composites for the PDO-index by positive (**a**)/negative (**b**) one standard deviation. All plots were generated using NCL.

Supplementary Fig. S12

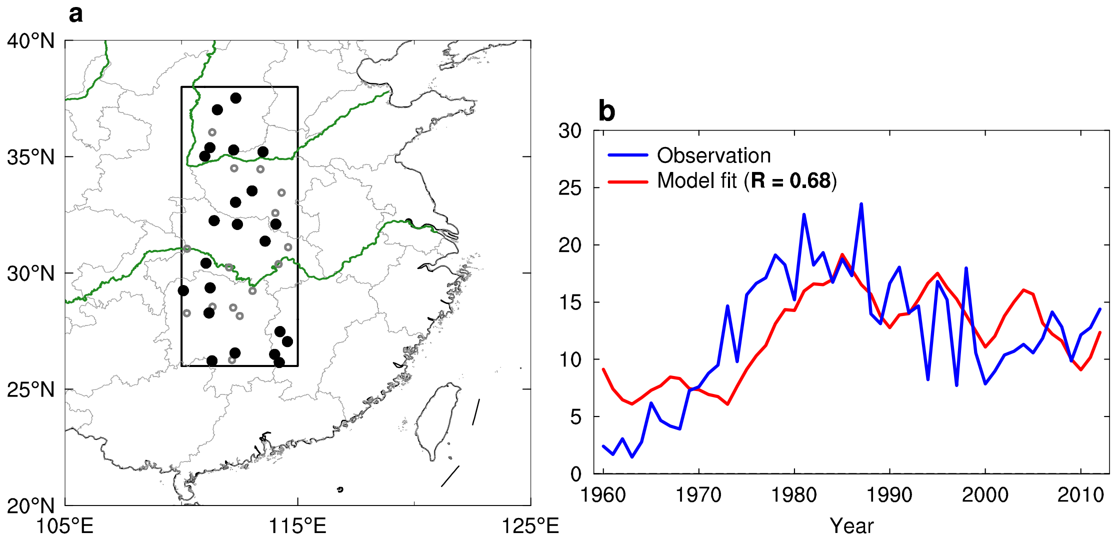

Supplementary Fig. S12. a, Locations of weather stations (total 36 stations) used for recording HD in central eastern China. Black solid dots (22 stations) denote the stations considered in Figure 6. **b**, Same as Figure 6, but showing the averaged wintertime HD time series for the 36 stations. All plots were generated using NCL. *Scientific Reports* remains neutral with regard to contested jurisdictional claims in published maps.

Supplementary Fig. S13

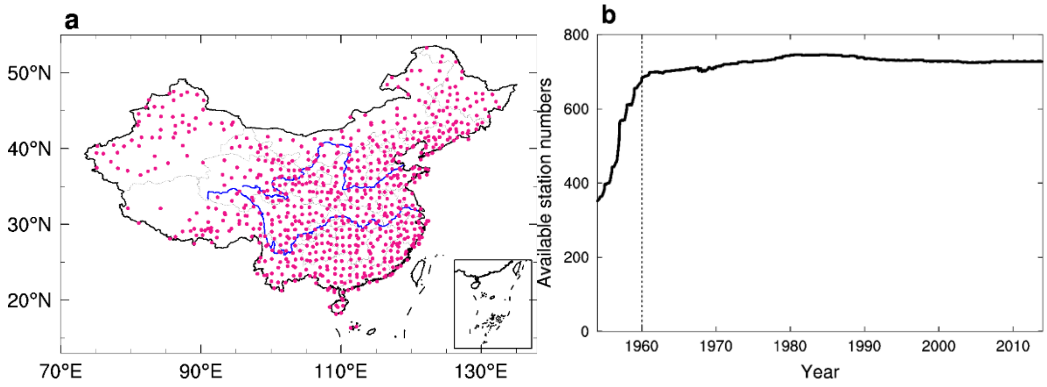

Supplementary Fig. S13. a, Locations of weather stations (total 756 stations) used to record haze days in China. **b**, Monthly number of stations recording haze days during 1954–2013. All plots were generated using NCL. *Scientific Reports* remains neutral with regard to contested jurisdictional claims in published maps.

Supplementary Fig. S14

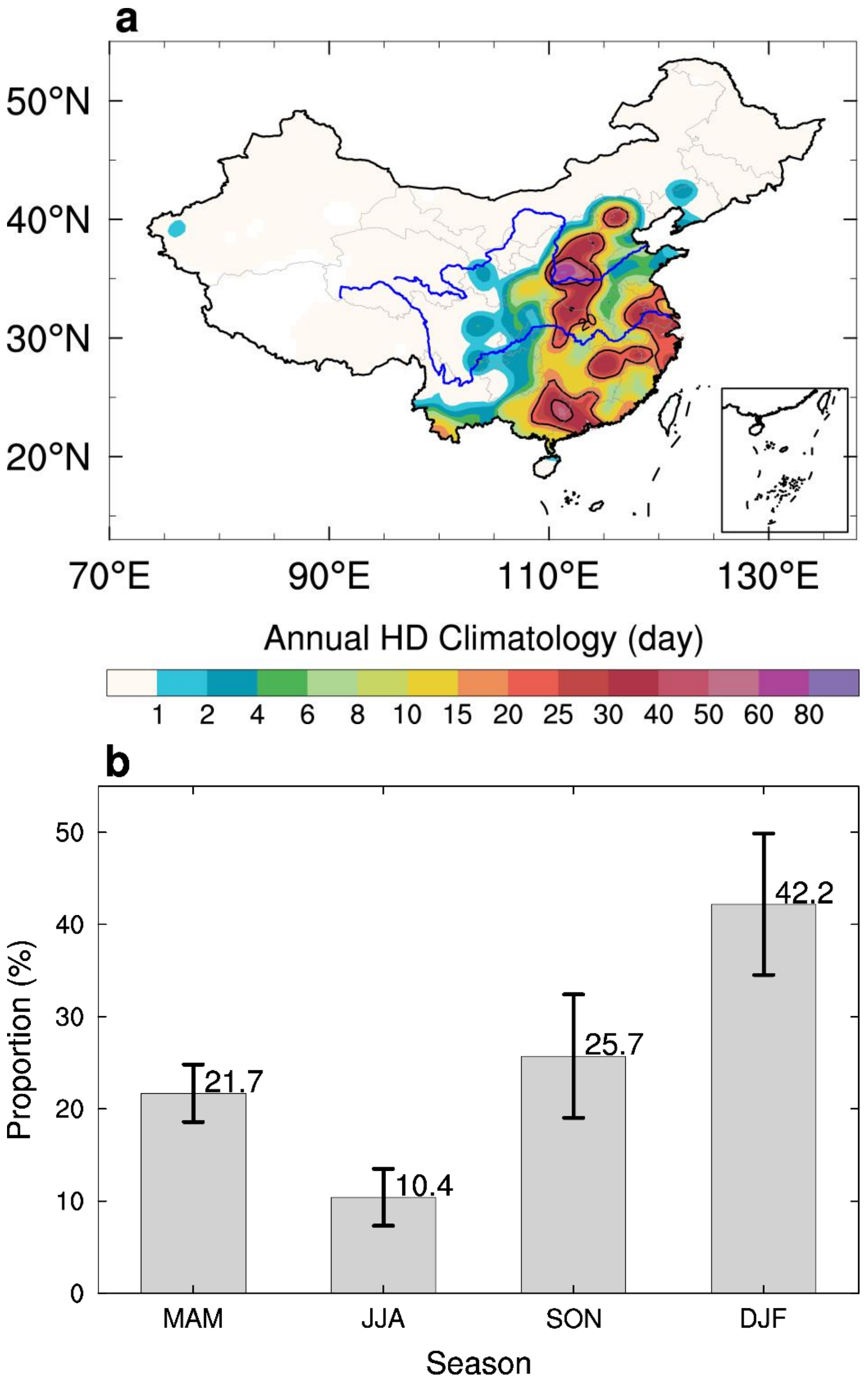

Supplementary Fig. S14. a, Spatial distribution of the climatology of the annual HD during 1981–2010. **b**, Seasonal mean proportions of the climatological HD at 756 stations and standard deviations during 1981–2010. All plots were generated using NCL. *Scientific Reports* remains neutral with regard to contested jurisdictional claims in published maps.

Supplementary Fig. S15

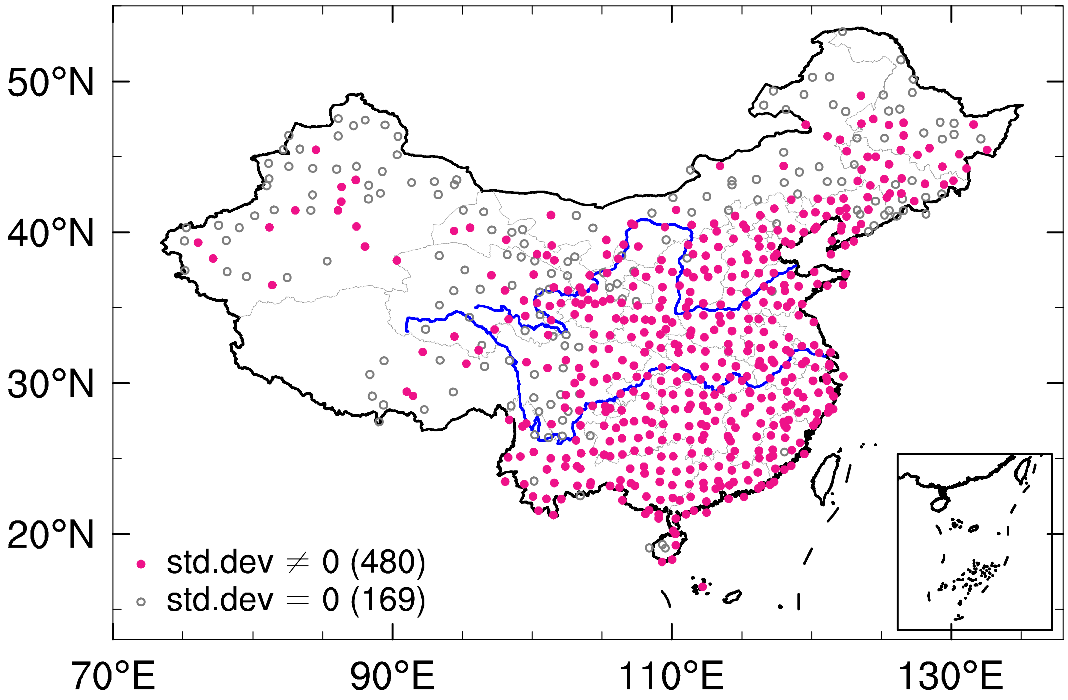

Supplementary Fig. S15. Locations of selected meteorological stations (pink dots) with wintertime HD records for 1959/1960–2012/2013. Grey open circles indicate stations for which the standard deviations of wintertime HD is equal to zero. Figures in brackets denote the numbers of stations. The plot was generated using NCL. *Scientific Reports* remains neutral with regard to contested jurisdictional claims in published maps.
